# Synthesis, Spectroscopic Characterization, Catalytic and Biological Activity of Oxidovanadium(V) Complexes with Chiral Tetradentate Schiff Bases

**DOI:** 10.3390/molecules28217408

**Published:** 2023-11-03

**Authors:** Grzegorz Romanowski, Justyna Budka, Iwona Inkielewicz-Stepniak

**Affiliations:** 1 Faculty of Chemistry, University of Gdansk, Wita Stwosza 63, PL-80308 Gdansk, Poland; 2 Department of Pharmaceutical Pathophysiology, Faculty of Pharmacy, Medical University of Gdansk, Dębinki 7, Building 27, PL-80211 Gdansk, Poland

**Keywords:** vanadium, Schiff base, catalysis, biological activity

## Abstract

New oxidovanadium(V) complexes, **VOL^1^**–**VOL^10^**, with chiral tetradentate Schiff bases obtained by monocondensation reaction of salicylaldehyde derivatives with 1*S*,2*S*-(+)-2-amino-1-(4-nitrophenyl)-1,3-propanediol. All complexes have been characterized using different spectroscopic methods, viz. IR, UV-Vis, circular dichroism, one- (^1^H, ^51^V) and two-dimensional (COSY, NOESY) NMR spectroscopy, and elemental analysis. Furthermore, the catalytic ability of all compounds in the epoxidation of styrene, cyclohexene, and its naturally occurring monoterpene derivatives, i.e., *S*(−)-limonene and (−)-α-pinene has also been studied, using two different oxidants, i.e., aqueous 30% H_2_O_2_ or *tert*-butyl hydroperoxide (TBHP). In addition, the biological properties of these chiral oxidovanadium(V) compounds, but also *cis*-dioxidomolybdenum(VI) complexes with the same chiral Schiff bases, were studied. Their cytotoxic and cytoprotective activity studies with the HT-22 hippocampal neuronal cells revealed a concentration-dependent effect in the range of 10–100 μM. Moreover, vanadium(V) complexes, in contrast to *cis*-dioxidomolybdenum(VI) compounds, demonstrated higher cytotoxicity and lack of cytoprotective ability against H_2_O_2_-induced cytotoxicity.

## 1. Introduction

Vanadium is one of the most important transition elements due to its wide biodiversity presence in terrestrial and marine environments, present among others in vanadium-dependent haloperoxidases in marine algae or terrestrial fungi and lichen [1,2,3], vanadium nitrogenases in Azotobacter genus [4] but also in compounds such as amavadin in the mushroom *Amanita muscaria* [5]. Research on the catalytic aspects of vanadium complexes as model compounds [1,6] was stimulated by their importance in various biological processes, but also as catalysts towards many organic transformations [7,8]. Chiral Schiff bases, also with salicylaldimine moiety, were found to be suitable for complexation to vanadium and, moreover, possess widespread applications [9,10]. Recently, especially useful for purpose are chiral *N*-salicyl-β-amino alcohol Schiff base ligands, considered “privileged ligands” [11], whose structural and electronic properties can be fine-tuned [12]. Vanadium(V) Schiff base complexes derived from amino alcohols have been successfully used as catalysts in the asymmetric alkynylation of aldehydes [13] and oxidation of organic sulfides to sulfoxides [13,14,15], oxidation of bromide [16], stereoselective synthesis of functionalized tetrahydrofurans [16,17], and epoxidation of alkenes [18,19]. Since epoxides have become important starting substrates for the synthesis of a wide variety of products, as for approved medicines and drug candidates, epoxidation of various olefins is recently one of the most widely studied reactions in organic chemistry [20]. The importance of epoxides mainly arises from the ring opening reactions, which further lead to the useful generation of new carbon-carbon bonds [21].

Various applications of vanadium complexes as prospective medicinal pharmaceutics have been extensively described so far [22]. Their several biological activities have been reported, such as antidiabetic [23,24], anti-inflammatory [25], antiparasitic [26], anticancer [27], but also therapeutic potential against HIV [28] and COVID-19 [29] viruses or tuberculosis [30] and pneumonia [31] bacteria. Recently, a growing interest of researchers has also been focused on their cytoprotective activity against oxidative damage in the HT-22 hippocampal neuronal cells [32,33]. Oxidative stress is a consistent component in the development of many neurodegenerative diseases, e.g., Alzheimer’s and Parkinson’s diseases, as well as stroke and brain trauma. To overcome oxidative stress in the brain, new innovative strategies to develop cytoprotective drugs are needed. An excellent model cell for studying the consequences of endogenous oxidative stress is the HT-22 hippocampal cells since it has been found that an important role in the mechanisms of neuronal susceptibility is played by mitochondria and energy metabolism [34].

**Figure 1:**
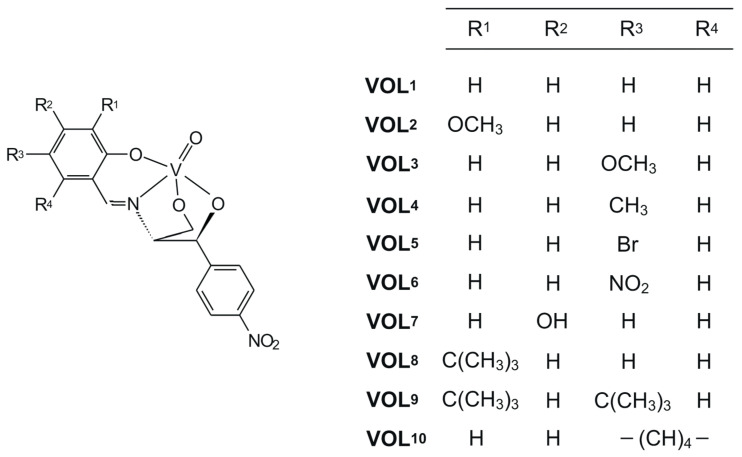
Structural formulae of oxidovanadium(V) complexes.

In the current paper, our efforts have been focused on new oxidovanadium(V) complexes with chiral tetradentate Schiff bases obtained using monocondensation of 1*S*,2*S*-(+)-2-amino-1-(4-nitrophenyl)-1,3-propanediol with salicylaldehyde derivatives, presented in Figure 1. All compounds have been characterized using various spectroscopic techniques, i.e., UV-Vis, circular dichroism, IR, and one- and two-dimensional NMR. Moreover, their catalytic abilities in the epoxidation of alkenes, i.e., styrene, cyclohexene, and its naturally occurring monoterpene derivatives, i.e., *S*(−)-limonene and (−)-α-pinene, in the presence of aqueous 30% H_2_O_2_ or tert-butyl hydroperoxide as the terminal oxidant, have been studied. Finally, the biological properties of these chiral oxidovanadium(V) compounds, but also *cis*-dioxidomolybdenum(VI) complexes with the same chiral Schiff bases, were investigated in relation to their cytotoxic properties in the range of 10–100 μM concentration and cytoprotective ability in the HT-22 hippocampal neuronal cells against exogenously generated oxidative damage using 30% hydrogen peroxide.

## 2. Results and Discussion

### 2.1. IR Spectra

Sharp and strong bands appear in the 964–982 cm^−1^ region in IR spectra of all chiral oxidovanadium(V) Schiff base complexes, **VOL^1^**–**VOL^10^**, which are close to values reported for similar vanadium(V) complexes and attributed to single V=O stretching vibrations [35]. Successful monocondensation during syntheses of the ligands is proved by characteristic strong imine C=N stretching vibrations at 1620–1632 cm^−1^, indicating the presence of chiral Schiff bases, also coordinated to the vanadium(V) ion [36]. The lack of any medium or strong intensity absorption bands in the range 3200–3700 cm^−1^, which could be assigned to O-H stretching modes, undoubtedly proves the deprotonation of phenolic and alcoholic hydroxyl groups. Moreover, the appearance of strong asymmetric and symmetric ν(C-O) vibrations at 1276–1316 and 1036–1109 cm^−1^, respectively, suggest the coordination of all alkoxy and aryloxy groups to vanadium atoms [Supplementary Figure S1]. Finally, all complexes exhibit characteristic asymmetric and symmetric ν(NO_2_) stretches at ca. 1520 and 1350 cm^−1^, respectively, which are present due to nitro substituent attached to the aromatic ring. 

### 2.2. Electronic and Circular Dichroism Spectra

Spectroscopic properties of all oxidovanadium(V) complexes have been studied by UV-Vis and circular dichroism techniques using dimethyl sulfoxide ACS spectrophotometric grade as a solvent. The UV-Vis spectra display strong absorption bands in the 314–354 nm region, which are assigned to an intraligand π-π* transitions and also a ligand-to-metal charge transfer (LMCT) transition from the phenolate oxygen p_π_ orbital to an empty d orbital of vanadium atom, which appears as the low-energy transitions appear between 426–477 nm [37]. Moreover, the spectrum of the chiral **VOL^10^** complex possessing naphthyl moiety displays two bands π-π* at 314 and 345 nm. The circular dichroism spectra revealed bands in the 263–288 and 302–322 nm regions, both with negative Cotton effects, and also in the 350–391 nm region, but with positive Cotton effect, of the same origin as electronic spectra.

### 2.3. NMR Measurements

All NMR spectra measured for oxidovanadium(V) complexes with tetradentate Schiff bases were recorded in DMSO-*d*_6_ solution, i.e., one-dimensional (^1^H, ^51^V) and two-dimensional (COSY and NOESY). The presence of azomethine proton signals in the ^1^H spectra of all complexes has proved a monocondensation reaction between all salicylaldehyde derivatives and chiral amino alcohol. Identification of all proton signals, a connection and proximity between all protons, and their assignments have been achieved using two-dimensional NMR experiments. For example, in the COSY spectrum of **VOL^5^**, a methine proton with a multiplet signal at 4.03 ppm unambiguously revealed its connection with two methylene protons, whose signals appeared as a doublet of doublets, and a second methine proton. These bonds have been confirmed using the presence of two cross-peaks between signals of both methylene protons attached to the hydroxyl group (4.10 and 4.24 ppm) and methine proton neighboring with a nitrogen atom (4.03 ppm). Furthermore, the latter methine proton cross-peak with strongly deshielded methine proton doublet at 6.02 ppm attached to the para-nitrophenyl group has also been found. On the other hand, in the NOESY spectrum, cross-peaks between the azomethine proton signal at 8.67 ppm with methine proton at 4.03 ppm, but also with doublet of doublets of methylene proton at 4.10 ppm, give information about their close spatial proximity [38]. Moreover, another cross-peak between the azomethine proton signal and the aromatic proton signal at 7.73 allows for the assignment of all signals belonging to aromatic ring protons. Finally, all the complexes exhibit single ^51^V NMR signals in the range −536.7 to −552.7 ppm, indicating their occurrence only in the single (presumably monomeric) form.

### 2.4. Catalytic Activity Studies

**Figure 2:**
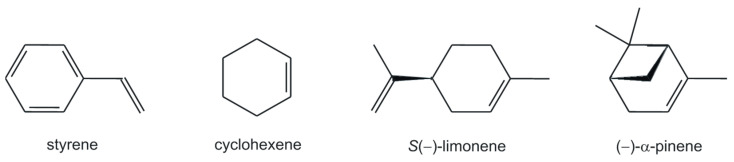
Substrates used for catalytic oxidation studies.

All oxidovanadium(V) complexes with chiral tetradentate Schiff bases, **VOL^1–10^**, have been studied for their catalytic abilities in epoxidation of olefins, i.e., styrene, cyclohexene and its naturally occurring monoterpene derivatives, i.e., *S*(−)-limonene and (−)-α-pinene (Figure 2). For the reactions performed with tert-butyl hydroperoxide (TBHP) as the terminal oxidant, 1,2-dichloroethane (DCE) was used as the solvent. On the other hand, when 30% aqueous H_2_O_2_ was employed for catalytic studies, no conversion was observed with DCE, and acetonitrile was chosen to avoid a biphasic system. Moreover, with regard to other common solvents like methanol, ethanol, toluene, chloroform, and methylene chloride, these solvents were found to be the most efficient. Moreover, for the two latter solvents, the poorest yields have been achieved, which may be caused by the lower reaction temperature for their reflux conditions. Higher reaction temperature, based on our observations in conversion and selectivity, has an overall benefit to achieving the best yields for performed epoxidation reactions, which required at least 5 h to reach completion. The same conclusions that better yields and reaction rates may be achieved with higher reaction temperatures have also been found in literature reports [39]. In our catalytic studies, we have also taken into account different reaction parameters in order to achieve suitable reaction conditions for a maximum reaction conversion. For this purpose, various oxidant molar ratios to the substrate (1:1, 2:1, 3:1, and 4:1), but also the amount of catalyst (0.5, 1, 2, and 3 mol% loadings) have been studied. The final observations have demonstrated that 1 mol% amounts of each catalyst were sufficient to run the epoxidation reactions with 3:1 molar ratio of both oxidants to all substrates. An increase in these ratios of catalysts or oxidants did not noticeably change the reaction rates. 

**Figure 3:**
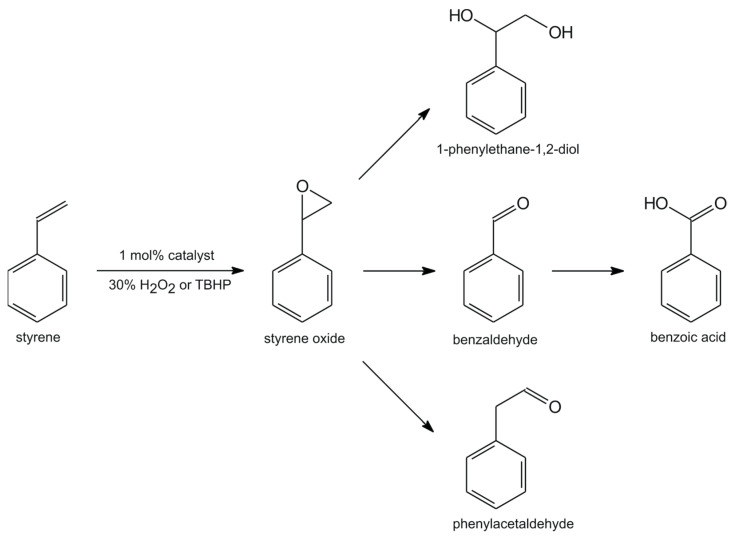
Various products of catalytic oxidation of styrene.

It was shown in our previous reports [40] that the oxidation of styrene can result in five oxidation products, i.e., styrene oxide, phenylacetaldehyde, 1-phenylethane-1,2-diol, benzaldehyde, and benzoic acid (Figure 3) using aqueous 30% H_2_O_2_ or TBHP as the oxygen sources and with catalytic amounts of different oxidovanadium(V) Schiff base complexes. In the first step of styrene conversion, styrene oxide can be formed. Further progress of this reaction is very fast, converting styrene oxide into benzaldehyde [41] via nucleophilic attack of an oxidant followed by the cleavage of the intermediate hydroperoxystyrene. Finally, benzaldehyde may also be further oxidized to benzoic acid. In alternative conversion, oxidative cleavage of the styrene side-chain double bond may be responsible for the direct formation of benzaldehyde via a radical mechanism. In the case of aqueous 30% H_2_O_2_, guilt for the very low conversion of styrene as a consequence of the decomposition of the catalyst may be shifted by the presence of water, which can cause the hydrolysis of styrene oxide and the formation of 1-phenylethane-1,2-diol. Phenylacetaldehyde may also be found as the product of styrene oxide isomerization.

The results of our catalytic studies of the epoxidation of styrene with the use of aqueous 30% H_2_O_2_ as the terminal oxidant, presented in Table 1 for **VOL^1–10^** complexes, have shown only low conversions (14–26%) and with benzaldehyde as the main product of this reaction. Similar results were found in our previous studies with vanadium(V) complexes with chiral tridentate Schiff bases as catalysts, also derived from amino alcohols [38,40]. When TBHP was used in a non-aqueous environment, the conversions of styrene significantly increased to 87%, and selectivity towards styrene oxide went up considerably. Both oxidants lead to the formation of styrene oxide and benzaldehyde as major products without any additional by-products.

On the other hand, as well as for reported earlier, [VO_2_(acac-ambmz)], [VO_2_(sal-ambmz)], [VO_2_(sal-aebmz)] [42], and [VO(hap-dahp)] complexes [43], styrene oxide is the most expected product, but its selectivity is really low (2–11%) and with high amounts of benzaldehyde, even 90% in case of [VO_2_(acac-aebmz)], and conversions from 51 to 78%. Unexpectedly, when TBHP was used as the terminal oxidant with catalytic amounts of these complexes, in contrast to **VOL^1–10^** catalysts, conversions significantly decreased to even 20%, but selectivity towards styrene oxide was slightly better, up to 47% in the case of [VO_2_(acac-ambmz)].

**Figure 4:**
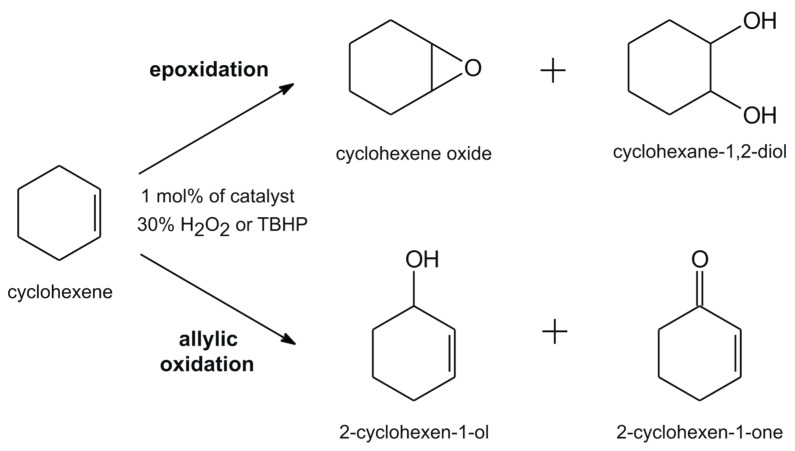
Possible epoxidation and allylic oxidation products of cyclohexene.

The other reaction studied in this paper was the oxidation of cyclohexene in the presence of catalytic amounts of **VOL^1–10^** complexes and with both oxygen sources, i.e., aqueous 30% H_2_O_2_ or *tert*-butyl hydroperoxide (TBHP). The reaction goes in two independent ways with cyclohexene oxide and, after its eventual hydrolysis, cyclohexene-1,2-diol as an epoxidation product, but also allylic oxidation products were observed, such as 2-cyclohexen-1-ol and after further oxidation resulting in the formation of 2-cyclohexen-1-one (Figure 4). When this catalytic reaction was performed with 30% H_2_O_2_ as the terminal oxidant, under the optimized reaction conditions, **VOL^1–10^** catalysts gave good up to 69% conversion. The percentage conversion of cyclohexene and the selectivities for the various reaction products is shown in Table 2. It is noteworthy that oxidovanadium(V) complex with tridentate Schiff base ligands, derived from 3-hydroxy-2 naphthohydrazide in the cyclohexene oxidation with H_2_O_2_ have shown higher conversion (92%), but significantly lower selectivity towards epoxide (29%) and 2-cyclohexen-1-one (8%), but high amounts of cyclohexen-1-ol (55%) [44]. Similar results have been reported in a series of vanadium(V) complexes with hydrazone ONO and NNS donor Schiff base ligands, i.e., [VO(μ_2_-OCH_3_)(L^1^)]_2_, [VO_2_(L^2^)]·H_2_O and [VO_2_(L^3^)] [45]. In contrast to H_2_O_2_, when TBHP was used, conversions were distinctly higher, up to 88%, but **VOL^1–10^** were less selective towards cyclohexene oxide, but higher amounts of 2-cyclohexene-1-one were noticed. Preferential attack of the activated C–H bond over the C=C bond may be responsible for the formation of the allylic oxidation products 2-cyclohexen-1-ol and 2-cyclohexen-1-one in higher selectivity [46].

Finally, the catalytic ability of all complexes was studied in the oxidation of mono- and bicyclic naturally occurring monoterpene derivatives of cyclohexene, giving analogous epoxidation and allylic oxidation products, i.e., *S*(−)-limonene and (−)-α-pinene. In comparison to cyclohexene, *S*(−)-limonene was oxidized by 30% H_2_O_2_ with very low conversions (Table 3), but epoxide was the main reaction product, up to 84%, and only small amounts of diepoxide as a by-product were obtained, due to the presence of additional exocyclic isopropenyl moiety. Interestingly, no allylic oxidation products were found. When TBHP was used as the terminal oxidant, distinctly better conversions were observed, up to 76%, but much more diepoxide was formed. The oxidation of (−)-α-pinene (Table 4) gave considerably lower conversions than in the case of *S*(−)-limonene with both oxidants, but distinctly better selectivity was achieved towards (−)-α-pinene oxide (up to 73% with TBHP).

### 2.5. Biological Studies

**Figure 5:**
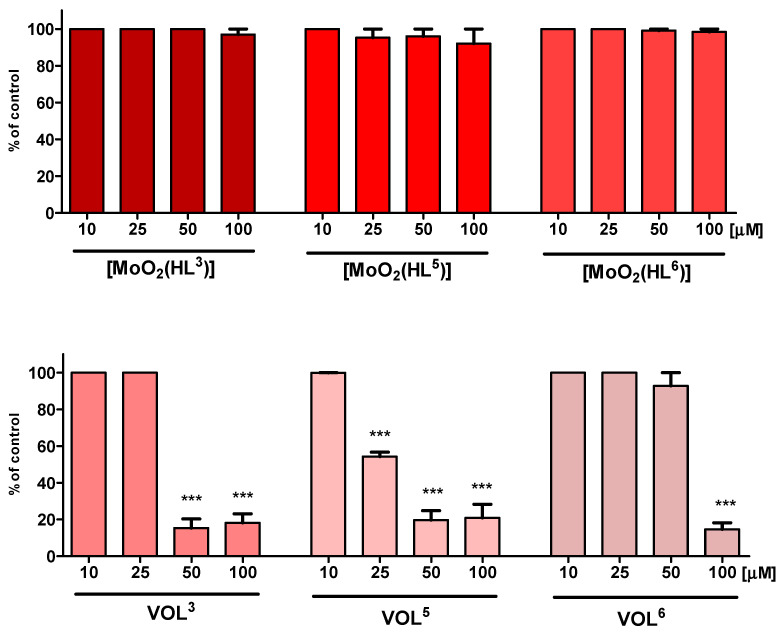
Viability of HT-22 cell line in MTT assay after 24 h exposure to test compounds. Data are expressed as mean ± SD values from three experiments. *** *p* < 0.001, as compared to control (untreated) cells.

**Figure 6:**
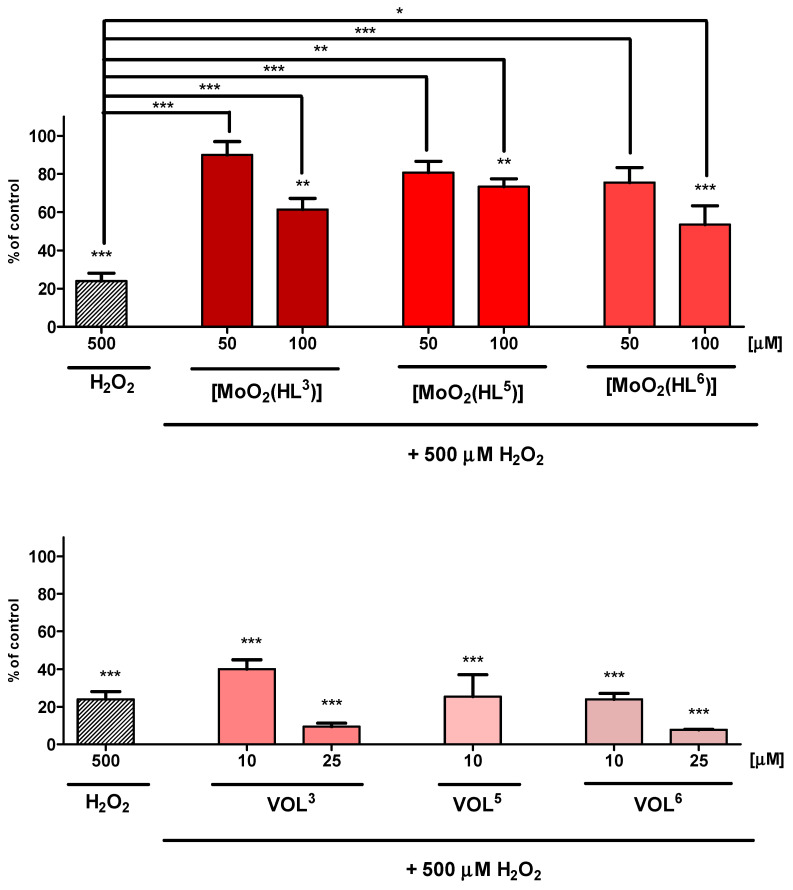
Viability of HT-22 cell line in MTT assay after 24 h exposure to test compounds and 500 µM H_2_O_2_. Data are expressed as mean ± SD values from three experiments. * *p* < 0.05, ** *p* < 0.01, *** *p* < 0.001, as compared to control (untreated) cells and to 500 µM H_2_O_2_.

Cytotoxic and cytoprotective activity studies of oxidovanadium(V) complexes, **VOL^3^**, **VOL^5^** and **VOL^6^**, and for comparison *cis*-dioxidomolybdenum(VI) complexes with the same chiral tetradentate Schiff base ligands [47], **[MoO_2_(HL^3^)]**, **[MoO_2_(HL^5^)]** and **[MoO_2_(HL^6^)]**, are presented on Figure 5 and Figure 6. 

The MTT test was used for assessing the cytotoxicity and the concentration-dependent effect at the mitochondrial level of the investigated compounds on the viability of the hippocampal neuronal cell line HT-22 in the 10–100 μM concentration range. Moreover, their cytoprotective activity was also studied against exogenously generated oxidative damage by hydrogen peroxide, which is the well-established precursor to reactive oxygen species (ROS) that are known to contribute to oxidative stress [48].

The comparison of the MTT test results between *cis*-dioxidomolybdenum(VI) and oxidovanadium(V) complexes has shown significant differences in cytotoxic effects depending on the presence of these metals in the coordination spheres of the same chiral Schiff base ligands (Figure 5). Surprisingly, all three *cis*-dioxidomolybdenum(VI) complexes, **[MoO_2_(HL^3^)]**, **[MoO_2_(HL^5^)]**, and **[MoO_2_(HL^6^)]**, have not revealed any distinct influence on the viability of HT-22 cells in their concentrations from 10 to 100 μM in the MTT tests. Furthermore, in the case of **[MoO_2_(HL^5^)]**, it has been noticed that 100 μM dose induces the highest decrease in viability of cells, but only to 92%. On the other hand, the results of MTT tests for **VOL^3^**, **VOL^5^**, and **VOL^6^** have disclosed very different results among each other and no significant influence on the viability of HT-22 cells at 25, 10, and even 50 μM dose, respectively, but their stronger cytotoxic effect in contrast to *cis*-dioxidomolybdenum(VI) complexes has been found. It is noteworthy that the weakest cytotoxic effect until 50 μM dose with over 90% cell viability has been revealed by **VOL^6^**, but at 100 μM, viability decreased dramatically below 20%. In the case of **VOL^3^**, no cytotoxic activity was observed until 25 μM dose, but this concentration in the case of oxidovandium(V) **VOL^5^** complex decreased the viability of HT-22 cells below 60%.

In the experiment assessing the cytoprotective activity of the investigated complexes, a 500 µM dose of 30% H_2_O_2_ was used to induce a cellular injury in the hippocampal cells. Treatment of HT-22 cells only with such concentration of H_2_O_2_ results in cellular death to about 24% after 24 h exposure due to oxidative stress (Figure 6). Cytoprotective properties of **[MoO_2_(HL^3^)]**, **[MoO_2_(HL^5^)]**, and **[MoO_2_(HL^6^)]**, which have shown no significant cytotoxicity from 10 to 100 μM concentrations, have been tested only in 50 and 100 μM doses. *Cis*-dioxidomolybdenum(VI) complexes exhibited statistically significant cytoprotective properties at the investigated concentrations. Increased viability was observed of HT-22 cells up to 90% for **[MoO_2_(HL^3^)]**, 81% for **[MoO_2_(HL^5^)]** and 75% for **[MoO_2_(HL^6^)]** at 50 μM dose. At higher concentrations of 100 μM, the complexes exhibited weaker cytoprotective effect and increased viability of hippocampal cells up to 54% for **[MoO_2_(HL^6^)]**, 61% for **[MoO_2_(HL^3^)]**, and 74% for **[MoO_2_(HL^5^)]**.

Screening of cytotoxic effects of **VOL^3^**, **VOL^5^**, and **VOL^6^** complexes, significant influence on viability of HT-22 cells at different concentrations has been observed. As it was concluded after comparison with **[MoO_2_(HL^3^)]**, **[MoO_2_(HL^5^)]**, and **[MoO_2_(HL^6^)]** compounds, vanadium(V) complexes have shown higher cytotoxicity and lack of cytoprotective ability against exogenously generated oxidative damage by hydrogen peroxide. Furthermore, their higher cytotoxicity than *cis*-dioxidomolybdenum(VI) compounds with the same chiral Schiff base ligand can be presumably caused by the forming peroxidovanadium(V) species with a strong harmful influence on biological cells [49]. On the other hand, such properties of vanadium(V) complexes make them potential drug candidates in targeted cancer therapies and emphasize their biological significance as promising anticancer lead compounds. Moreover, various vanadium compounds as anticancer agents are of growing interest, showing various beneficial properties for chemotherapy, i.e., strong cytotoxicity or antimetastatic activity [50].

**Figure 7:**
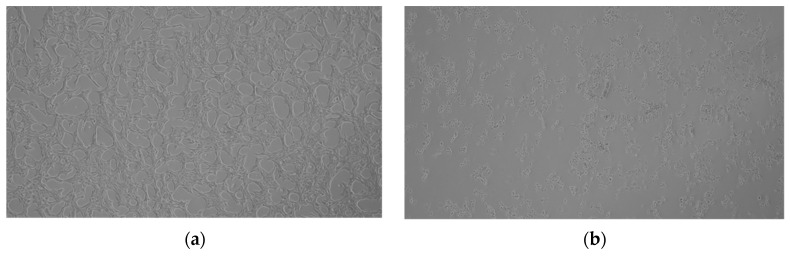

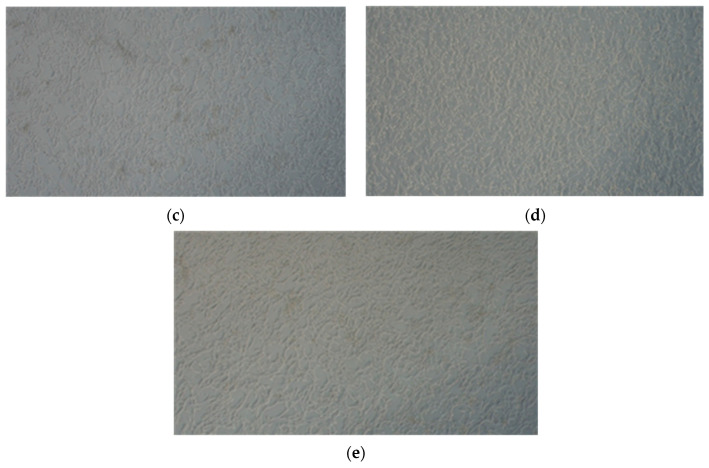
HT-22 cell morphology: (**a**) control cells and after treatment with: (**b**) 500 µM H_2_O_2_; (**c**) 50 µM **[MoO_2_(HL^3^)]** and 500 µM H_2_O_2_, (**d**) 50 µM **[MoO_2_(HL^5^)]**, and 500 µM H_2_O_2_, (**e**) 50 µM **[MoO_2_(HL^6^)]** and 500 µM H_2_O_2_.

Finally, microscopic images have shown significant morphological changes in HT-22 cells after the exposure to 30% H_2_O_2_ (500 µM), which have been characterized, as compared with control cell (Figure 7a), by irregular shapes and cell shrinkage (Figure 7b) and the cells pre-treated with 50 μM *cis*-dioxidomolybdenum(VI) complexes exhibiting the best cytoprotective activity (Figure 7c–e).

## 3. Materials and Methods

### 3.1. Measurements

All reagents and solvents were used without further purification and obtained from commercial sources. For elemental analyses, a Carlo Erba MOD 1106 instrument was employed. All electronic spectra and circular dichroism were measured using spectrophotometric grade DMSO and were recorded, respectively, on a Perkin-Elmer LAMBDA 18 spectrophotometer and a Jasco J-815 spectropolarimeter. IR spectra were run on a Bruker IFS 66 from solid samples as KBr pellets. Bruker AVANCE III 700 MHz spectrometer was used for obtaining NMR spectra using TMS as a reference and DMSO-*d*_6_ as a solvent. Progress of all catalytic reactions was measured on Shimadzu GC-2025 gas chromatograph with FID detector on a Zebron ZB-5 capillary column (30 m × 0.25 mm × 0.25 mm), and GC-MS instrument Shimadzu GCMS-QP2010 SE (Shimadzu Europa GmbH, Duisburg, Germany) was used for identification of the reaction products.

### 3.2. Synthesis of Oxidovanadium(V) Complexes

The following procedure for synthesis of all complexes was employed. To 10 mL of methanol, 1 mmol of 1*S*,2*S*-(+)-2-amino-1-(4-nitrophenyl)-1,3-propanediol was added, following with further addition of 1 mmol of aromatic *o*-hydroxyaldehyde, i.e., salicylaldehyde, 3-methoxysalicylaldehyde, 5-methoxysalicylaldehyde, 5-methylsalicylaldehyde, 5-bromosalicylaldehyde, 5-nitrosalicylaldehyde, 4-hydroxysalicylaldehyde, 3-*tert*-butylsalicylaldehyde, 3,5-di-*tert*-butylsalicylaldehyde or 2-hydroxy-1-naphthaldehyde dissolved in 10 mL of MeOH and reaction mixture was heated with stirring under reflux for 1 h. Next, vanadium(V) oxytripropoxide (1 mmol) was added as a metal precursor, and such reaction mixture was stirred under reflux for the next 2 h. After cooling was obtained, brown solids were separated, filtered off, and washed several times with cold MeOH.

***VOL^1^***: Yield 80%. *Anal.* Calc. for C_16_H_13_N_2_O_6_V: C, 50.5; H, 3.5; N, 7.4. Found: C, 50.6; H, 3.6; N, 7.3%. IR (KBr, cm^−1^): 1621 (ν_C=N_); 1600 (ν_C=C_); 1520, 1395 (ν_NO2_); 1285, 1069 (ν_C-O_); 972 (ν_V=O_). UV-Vis spectrum in DMSO [λ_max_ (nm), ε (M^−1^ cm^−1^)]: 322 (9350), 445 (1230). CD spectrum in DMSO [λ_max_ (nm), Δε (M^−1^ cm^−1^)]: 270 (−1.96), 309 (−1.32), 367 (2.69). ^1^H NMR (DMSO-*d*_6_, ppm): 8.69 (1H, s) (azomethine); 8.25 (2H, d, ^3^*J* = 8.5 Hz), 7.67 (2H, d, ^3^*J* = 8.5 Hz), 7.55 (2H, m), 6.98 (2H, m) (aromatic); 5.99 (1H, d, ^3^*J* = 4.4 Hz), 4.09 (1H, m) (methine); 4.30 (1H, dd, ^3^*J* = 11.6 Hz, ^4^*J* = 4.5 Hz), 4.22 (1H, dd, ^3^*J* = 12.0 Hz, ^4^*J* = 7.6 Hz) (methylene). ^51^V NMR (DMSO-*d*_6_, ppm): −541.9. 

***VOL^2^***: Yield 86%. *Anal.* Calc. for C_17_H_15_N_2_O_7_V: C, 49.8; H, 3.7; N, 6.8. Found: C, 49.7; H, 3.6; N, 6.9%. IR (KBr, cm^−1^): 1623 (ν_C=N_); 1600 (ν_C=C_); 1521, 1346 (ν_NO2_); 1290, 1080 (ν_C-O_); 978 (ν_V=O_). UV-Vis spectrum in DMSO [λ_max_ (nm), ε (M^−1^ cm^−1^)]: 338 (4520), 452 (1140). CD spectrum in DMSO [λ_max_ (nm), Δε (M^−1^ cm^−1^)]: 272 (−1.83), 318 (−1.24), 379 (2.39). ^1^H NMR (DMSO-*d*_6_, ppm): 8.67 (1H, s) (azomethine); 8.24 (2H, d, ^3^*J* = 8.5 Hz), 7.66 (2H, d, ^3^*J* = 8.5 Hz), 7.22 (1H, d, ^3^*J* = 7.6 Hz), 7.14 (1H, d, ^3^*J* = 7.6 Hz), 6.92 (1H, t, ^3^*J* = 9.2 Hz) (aromatic); 5.97 (1H, d, ^3^*J* = 3.9 Hz), 4.08 (1H, m) (methine); 4.29 (1H, dd, ^3^*J* = 11.6 Hz, ^4^*J* = 3.9 Hz), 4.22 (1H, dd, ^3^*J* = 11.6 Hz, ^4^*J* = 7.4 Hz) (methylene); 3.93 (3H, s) (methoxy). ^51^V NMR (DMSO-*d*_6_, ppm): −537.6.

***VOL^3^***: Yield 92%. *Anal.* Calc. for C_17_H_15_N_2_O_7_V: C, 49.8; H, 3.7; N, 6.8. Found: C, 49.8; H, 3.8; N, 6.8%. IR (KBr, cm^−1^): 1629 (ν_C=N_); 1607 (ν_C=C_); 1519, 1346 (ν_NO2_); 1276, 1036 (ν_C-O_); 965 (ν_V=O_). UV-Vis spectrum in DMSO [λ_max_ (nm), ε (M^−1^ cm^−1^)]: 348 (4680), 477 (1190). CD spectrum in DMSO [λ_max_ (nm), Δε (M^−1^ cm^−1^)]: 278 (−1.65), 322 (−1.13), 387 (2.24). ^1^H NMR (DMSO-*d*_6_, ppm): 8.76 (1H, s) (azomethine); 8.22 (2H, d, ^3^*J* = 8.5 Hz), 7.63 (2H, d, ^3^*J* = 8.5 Hz), 7.19 (1H, dd, ^3^*J* = 8.3 Hz, ^4^*J* = 3.1 Hz), 7.07 (1H, d, ^3^*J* = 3.1 Hz), 6.89 (1H, d, ^3^*J* = 8.9 Hz) (aromatic); 5.97 (1H, d, ^3^*J* = 3.9 Hz), 4.07 (1H, m) (methine); 4.28 (1H, dd, ^3^*J* = 11.6 Hz, ^4^*J* = 3.9 Hz), 4.20 (1H, dd, ^3^*J* = 11.6 Hz, ^4^*J* = 7.4 Hz) (methylene); 3.79 (3H, s) (methoxy). ^51^V NMR (DMSO-*d*_6_, ppm): −536.7.

***VOL^4^***: Yield 90%. *Anal.* Calc. for C_17_H_15_N_2_O_6_V: C, 51.8; H, 3.8; N, 7.1. Found: C, 51.9; H, 3.7; N, 7.0%. IR (KBr, cm^−1^): 1625 (ν_C=N_); 1602 (ν_C=C_); 1521, 1346 (ν_NO2_); 1287, 1069 (ν_C-O_); 982 (ν_V=O_). UV-Vis spectrum in DMSO [λ_max_ (nm), ε (M^−1^ cm^−1^)]: 336 (5570), 454 (1250). CD spectrum in DMSO [λ_max_ (nm), Δε (M^−1^ cm^−1^)]: 271 (−1.60), 309 (−1.10), 375 (2.26). ^1^H NMR (DMSO-*d*_6_, ppm): 8.62 (1H, s) (azomethine); 8.24 (2H, d, ^3^*J* = 8.5 Hz), 7.64 (2H, d, ^3^*J* = 8.5 Hz), 7.40 (1H, d, ^3^*J* = 8.6 Hz), 7.33 (1H, d, ^3^*J* = 2.4 Hz), 6.88 (1H, d, ^3^*J* = 8.6 Hz) (aromatic); 5.94 (1H, d, ^3^*J* = 3.9 Hz), 4.08 (1H, m) (methine); 4.30 (1H, dd, ^3^*J* = 11.8 Hz, ^4^*J* = 4.9 Hz), 4.24 (1H, dd, ^3^*J* = 11.8 Hz, ^4^*J* = 7.6 Hz) (methylene); 2.32 (3H, s) (methyl). ^51^V NMR (DMSO-*d*_6_, ppm): −539.0.

***VOL^5^***: Yield 87%. *Anal.* Calc. for BrC_16_H_12_N_2_O_6_V: C, 41.9; H, 2.6; N, 6.1. Found: C, 41.8; H, 2.7; N, 6.0%. IR (KBr, cm^−1^): 1626 (ν_C=N_); 1604 (ν_C=C_); 1521, 1346 (ν_NO2_); 1283, 1072 (ν_C-O_); 976 (ν_V=O_). UV-Vis spectrum in DMSO [λ_max_ (nm), ε (M^−1^ cm^−1^)]: 340 (5420), 448 (1270). CD spectrum in DMSO [λ_max_ (nm), Δε (M^−1^ cm^−1^)]: 277 (−1.31), 304 (−1.05), 373 (2.15). ^1^H NMR (DMSO-*d*_6_, ppm): 8.67 (1H, s) (azomethine); 8.26 (2H, d, ^3^*J* = 8.5 Hz), 7.73 (1H, ov), 7.71 (2H, d, ^3^*J* = 8.5 Hz), 7.61 (1H, dd, ^3^*J* = 8.9 Hz, ^4^*J* = 2.4 Hz), 6.90 (1H, d, ^3^*J* = 8.9 Hz) (aromatic); 6.04 (1H, d, ^3^*J* = 5.6 Hz), 4.03 (1H, m) (methine); 4.24 (1H, dd, ^3^*J* = 11.9 Hz, ^4^*J* = 3.7 Hz), 4.10 (1H, dd, ^3^*J* = 11.9 Hz, ^4^*J* = 7.3 Hz) (methylene). ^51^V NMR (DMSO-*d*_6_, ppm): −542.1.

***VOL^6^***: Yield 81%. *Anal.* Calc. for C_16_H_12_N_3_O_8_V: C, 45.2; H, 2.8; N, 9.9. Found: C, 45.1; H, 2.9; N, 10.0%. IR (KBr, cm^−1^): 1632 (ν_C=N_); 1607 (ν_C=C_); 1520, 1344 (ν_NO2_); 1316, 1100 (ν_C-O_); 972 (ν_V=O_). UV-Vis spectrum in DMSO [λ_max_ (nm), ε (M^−1^ cm^−1^)]: 343 (8210), 426 (3400). CD spectrum in DMSO [λ_max_ (nm), Δε (M^−1^ cm^−1^)]: 275 (−1.23), 311 (−0.94), 354 (1.89). ^1^H NMR (DMSO-*d*_6_, ppm): 8.85 (1H, s) (azomethine); 8.55 (1H, d, ^3^*J* = 2.5 Hz), 8.35 (1H, dd, ^3^*J* = 9.1 Hz, ^4^*J* = 2.5 Hz), 8.29 (2H, d, ^3^*J* = 8.5 Hz), 7.78 (2H, d, ^3^*J* = 8.5 Hz), 7.03 (1H, d, ^3^*J* = 9.1 Hz) (aromatic); 6.24 (1H, d, ^3^*J* = 6.5 Hz), 3.96 (1H, m) (methine); 4.21 (1H, dd, ^3^*J* = 10.8 Hz, ^4^*J* = 3.2 Hz), 4.01 (1H, dd, ^3^*J* = 10.8 Hz, ^4^*J* = 7.1 Hz) (methylene). ^51^V NMR (DMSO-*d*_6_, ppm): −540.4.

***VOL^7^***: Yield 83%. *Anal.* Calc. for C_16_H_13_N_2_O_7_V: C, 48.5; H, 3.3; N, 7.1. Found: C, 48.5; H, 3.4; N, 7.1%. IR (KBr, cm^−1^): 1630 (ν_C=N_); 1606 (ν_C=C_); 1542, 1350 (ν_NO2_); 1293, 1109 (ν_C-O_); 974 (ν_V=O_). UV-Vis spectrum in DMSO [λ_max_ (nm), ε (M^−1^ cm^−1^)]: 354 (5830), 458 (1360). CD spectrum in DMSO [λ_max_ (nm), Δε (M^−1^ cm^−1^)]: 270 (−1.69), 312 (−1.17), 367 (2.36). ^1^H NMR (DMSO-*d*_6_, ppm): 9.72 (1H, s) (hydroxyl); 8.46 (1H, s) (azomethine); 8.25 (2H, d, ^3^*J* = 8.5 Hz), 7.62 (2H, d, ^3^*J* = 8.5 Hz), 7.50 (1H, d, ^3^*J* = 8.6 Hz), 6.46 (1H, dd, ^3^*J* = 8.6 Hz, ^4^*J* = 2.2 Hz), 6.29 (1H, d, ^3^*J* = 2.2 Hz) (aromatic); 5.91 (1H, d, ^3^*J* = 3.9 Hz), 4.07 (1H, m) (methine); 4.30 (1H, dd, ^3^*J* = 11.6 Hz, ^4^*J* = 3.5 Hz), 4.19 (1H, dd, ^3^*J* = 11.6 Hz, ^4^*J* = 7.4 Hz) (methylene). ^51^V NMR (DMSO-*d*_6_, ppm): −537.5.

***VOL^8^***: Yield 86%. *Anal.* Calc. for C_20_H_21_N_2_O_6_V: C, 55.1; H, 4.9; N, 6.4. Found: C, 55.0; H, 4.9; N, 6.3%. IR (KBr, cm^−1^): 1624 (ν_C=N_); 1591 (ν_C=C_); 1521, 1346 (ν_NO2_); 1289, 1084 (ν_C-O_); 975 (ν_V=O_). UV-Vis spectrum in DMSO [λ_max_ (nm), ε (M^−1^ cm^−1^)]: 349 (6670), 449 (1440). CD spectrum in DMSO [λ_max_ (nm), Δε (M^−1^ cm^−1^)]: 263 (−1.37), 315 (−0.67), 371 (2.20). ^1^H NMR (DMSO-*d*_6_, ppm): 8.68 (1H, s) (azomethine); 8.24 (2H, d, ^3^*J* = 8.5 Hz), 7.66 (2H, d, ^3^*J* = 8.5 Hz), 7.60 (1H, d, ^3^*J* = 7.6 Hz), 7.41 (1H, d, ^3^*J* = 7.6 Hz), 6.94 (1H, t, ^3^*J* = 8.2 Hz) (aromatic); 5.93 (1H, d, ^3^*J* = 3.7 Hz), 4.12 (1H, m) (methine); 4.31 (1H, dd, ^3^*J* = 11.5 Hz, ^4^*J* = 5.1 Hz), 4.25 (1H, dd, ^3^*J* = 11.5 Hz, ^4^*J* = 7.6 Hz) (methylene); 1.51 (9H, s) (*tert*-butyl). ^51^V NMR (DMSO-*d*_6_, ppm): −552.7.

***VOL^9^***: Yield 82%. *Anal.* Calc. for C_24_H_29_N_2_O_6_V: C, 58.5; H, 5.9; N, 5.7. Found: C, 58.7; H, 5.8; N, 5.7%. IR (KBr, cm^−1^): 1620 (ν_C=N_); 1598 (ν_C=C_); 1521, 1346 (ν_NO2_); 1296, 1080 (ν_C-O_); 964 (ν_V=O_). UV-Vis spectrum in DMSO [λ_max_ (nm), ε (M^−1^ cm^−1^)]: 351 (6320), 451 (1330). CD spectrum in DMSO [λ_max_ (nm), Δε (M^−1^ cm^−1^)]: 265 (−1.36), 302 (−1.12), 374 (2.13). ^1^H NMR (DMSO-*d*_6_, ppm): 8.70 (1H, s) (azomethine); 8.26 (2H, d, ^3^*J* = 8.5 Hz), 7.67 (2H, d, ^3^*J* = 8.5 Hz), 7.49 (1H, d, ^3^*J* = 2.7 Hz), 7.38 (1H, d, ^3^*J* = 2.7 Hz) (aromatic); 5.96 (1H, d, ^3^*J* = 3.7 Hz), 4.14 (1H, m) (methine); 4.29 (1H, dd, ^3^*J* = 11.7 Hz, ^4^*J* = 5.2 Hz), 4.23 (1H, dd, ^3^*J* = 11.7 Hz, ^4^*J* = 7.6 Hz) (methylene); 1.53 (9H, s), 1.41 (9H, s) (*tert*-butyl). ^51^V NMR (DMSO-*d*_6_, ppm): −547.3.

***VOL^10^***: Yield 88%. *Anal.* Calc. for C_20_H_15_N_2_O_6_V: C, 55.8; H, 3.5; N, 6.5. Found: C, 55.9; H, 3.4; N, 6.6%. IR (KBr, cm^−1^): 1621 (ν_C=N_); 1605 (ν_C=C_); 1519, 1344 (ν_NO2_); 1296, 1072 (ν_C-O_); 980 (ν_V=O_). UV-Vis spectrum in DMSO [λ_max_ (nm), ε (M^−1^ cm^−1^)]: 314 (8430), 345 (3660), 452 (1320). CD spectrum in DMSO [λ_max_ (nm), Δε (M^−1^ cm^−1^)]: 288 (−2.52), 319 (−3.92), 393 (1.29). ^1^H NMR (DMSO-*d*_6_, ppm): 9.59 (1H, s) (azomethine); 8.32 (1H, d, ^3^*J* = 8.5 Hz), 8.25 (2H, d, ^3^*J* = 8.5 Hz), 8.07 (1H, d, ^3^*J* = 8.6 Hz), 7.85 (1H, d, ^3^*J* = 7.9 Hz), 7.69 (2H, d, ^3^*J* = 8.5 Hz), 7.59 (1H, t, ^3^*J* = 8.6 Hz), 7.41 (1H, t, ^3^*J* = 8.6 Hz), 7.20 (1H, d, ^3^*J* = 9.2 Hz) (aromatic); 5.98 (1H, d, ^3^*J* = 4.1 Hz), 3.95 (1H, m) (methine); 4.34 (1H, dd, ^3^*J* = 11.5 Hz, ^4^*J* = 4.3 Hz), 4.28 (1H, dd, ^3^*J* = 11.5 Hz, ^4^*J* = 7.4 Hz) (methylene). ^51^V NMR (DMSO-*d*_6_, ppm): −541.1.

### 3.3. Catalytic Activity

Epoxidation of two alkenes, i.e., styrene and cyclohexene, and monoterpenes, i.e., *S*(−)-limonene and (−)-α-pinene, has been studied in the presence of all chiral oxidovanadium(V) complexes using, as the terminant oxidant, aqueous 30% H_2_O_2_ or 5.5 M solution of *tert*-butyl hydroperoxide (TBHP) in decane. After optimization of the reaction conditions, with different amounts of catalysts and oxidants, all reactions were run using 1:100:200 molar ratio of catalyst, substrate, and oxidant, respectively, at 80 °C with in 1,2-dichloroethane (DCE) as the solvent for TBHP and acetonitrile for H_2_O_2_. The reaction progress was monitored using GC, and the yields were recorded as GC yield based on the starting substrate. The identity of oxidation products was confirmed using GC-MS.

### 3.4. Biological Activity

#### 3.4.1. The Cell Culture

The authors want to thank Prof. Tilman Grune from Friedrich Schiller University in Jena (Germany) for a kind gift of the mouse hippocampal neuronal cell line HT-22. Dulbecco’s Modified Eagle’s medium supplemented with 10% fetal bovine serum (FBS), 1.5 g/L NaHCO_3_, 4 mM l-glutamine, 1000 mg/L glucose, 1 mM sodium pyruvate, 100 U/mL penicillin and 100 µg/mL streptomycin was employed to culture HT-22 cell line. Cultures were maintained at 37 °C in a humidified atmosphere of 5% CO_2_. Cells were regularly split and subcultured up to 80–90% confluence (2–3 times per week).

#### 3.4.2. Treatments

Working solutions at 10, 25, 50, and 100 µM concentrations were prepared in serum-free medium DMEM every time before adding to the cell line. The evaluation of the viability and cytoprotective action of investigated compounds on HT-22 hippocampal neuronal cells was tested by using mitochondrial dehydrogenase activity assay, MTT (Sigma Aldrich, Darmstadt, Germany), after 24 h incubation.

#### 3.4.3. MTT Assay

HT-22 cells were passaged at 8 × 10^3^ cells in a well of a 96-well plate. The plates were incubated under standard conditions in an incubator for 24 h. After 24 h, HT-22 cells were treated with selected concentrations that were not cytotoxic to cells for 24 h. In experiments studying the cytoprotective effects, investigated compounds were added 1 h before treatment with 30% H_2_O_2_ (500 µM) for 24 h. MTT assay was performed by adding a premixed optimized dye solution triazole blue formazan to culture wells. After incubation, the medium was removed, and the formed formazan crystals were dissolved by adding 100 µL DMSO into each well. Next, absorbance was measured at length waves 570 nm and 660 nm (reference value) in a microplate reader.

#### 3.4.4. Microscopy

HT-22 cells were seeded in 12-well plates. The plate was stored under standard conditions for 24 h. After 24 h, test compounds were added to HT-22 cells one hour prior to treatment with 30% H_2_O_2_ (500 µM) and incubated for 24 h. Images were then taken with an Olympus microscope at 1 × 4 magnification.

## 4. Conclusions

In our paper, we described new oxidovanadium(V) complexes with chiral tetradentate Schiff bases, **VOL^1^**–**VOL^10^**, which have been synthesized by monocondensation reaction of salicylaldehyde and its derivatives with 1*S*,2*S*-(+)-2-amino-1-(4-nitrophenyl)-1,3-propanediol. All compounds have been characterized using different spectroscopic methods, *viz*. IR, UV-Vis, circular dichroism, one- (^1^H, ^51^V) and two-dimensional (COSY, NOESY) NMR spectroscopy, and elemental analysis. 

Furthermore, these oxidovanadium(V) complexes, **VOL^1^**–**VOL^10^**, have also been employed as catalysts in the epoxidation reactions for testing their catalytic abilities. For this purpose, model olefinic substrates, i.e., styrene, cyclohexene, and its naturally occurring monoterpenes, i.e., *S*(−)-limonene and (−)-α-pinene, have been studied using the oxygen source two oxidants, i.e., aqueous 30% H_2_O_2_ or *tert*-butyl hydroperoxide. Conversion of all these olefins was distinctly lower when 30% H_2_O_2_ was used, but due to the strong oxidizing nature of hydrogen peroxide and the presence of a significant amount of water, which could be responsible for the decomposition of the catalyst and also the hydrolysis of epoxides. On the other hand, *tert*-butyl hydroperoxide (TBHP) in a non-aqueous environment proved to be a very good oxidizing agent, giving excellent conversions of substrates with epoxide as the main product.

The biological studies, based on an assessment of mitochondrial activity in MTT tests, have revealed cytoprotective activity of *cis*-dioxidomolybdenum(VI) and oxidovanadium(V) complexes with the same chiral Schiff base ligands towards the hippocampal neuronal cell line HT-22 against the oxidative damage generated exogenously by using hydrogen peroxide. The comparison of the MTT test results between *cis*-dioxidomolybdenum(VI) and oxidovanadium(V) complexes has shown significantly higher cytotoxic activity in lower concentrations for the latter compounds, which strongly depends on the concentration used. The cytoprotective activity experiments with a 500 µM dose of 30% H_2_O_2_ have shown their strong influence on viability at even 100 µM dose in the case of *cis*-dioxidomolybdenum(VI) compounds. Unfortunately, all investigated oxidovanadium(V) complexes, from 10 to 100 μM concentrations, have not exhibited any cytoprotective effect against the oxidative damage caused by hydrogen peroxide.

## Figures and Tables

**Table 1:** Epoxidation of styrene in the presence of oxidovanadium(V) Schiff base complexes as catalysts.

Entry	Catalyst	Yield (%)	Oxidant	Styrene Oxide (%)	Benzaldehyde (%)
1	**VOL^1^**	14	H_2_O_2_	7	93
2	**VOL^2^**	20	H_2_O_2_	11	89
3	**VOL^3^**	16	H_2_O_2_	5	95
4	**VOL^4^**	22	H_2_O_2_	8	92
5	**VOL^5^**	21	H_2_O_2_	10	90
6	**VOL^6^**	22	H_2_O_2_	6	94
7	**VOL^7^**	23	H_2_O_2_	12	88
8	**VOL^8^**	20	H_2_O_2_	8	92
9	**VOL^9^**	18	H_2_O_2_	11	89
10	**VOL^10^**	26	H_2_O_2_	7	93
11	**VOL^1^**	84	TBHP	31	69
12	**VOL^2^**	74	TBHP	17	83
13	**VOL^3^**	87	TBHP	29	71
14	**VOL^4^**	85	TBHP	28	72
15	**VOL^5^**	63	TBHP	39	61
16	**VOL^6^**	67	TBHP	38	62
17	**VOL^7^**	61	TBHP	32	68
18	**VOL^8^**	81	TBHP	27	73
19	**VOL^9^**	84	TBHP	37	63
20	**VOL^10^**	68	TBHP	22	78

**Table 2:** Epoxidation of cyclohexene in the presence of oxidovanadium(V) Schiff base complexes as catalysts.

Entry	Catalyst	Yield (%)	Oxidant	Epoxide (%)	Alcohol (%)	Ketone (%)
1	**VOL^1^**	64	H_2_O_2_	45	38	17
2	**VOL^2^**	45	H_2_O_2_	60	40	-
3	**VOL^3^**	66	H_2_O_2_	56	34	10
4	**VOL^4^**	41	H_2_O_2_	58	33	9
5	**VOL^5^**	45	H_2_O_2_	70	14	16
6	**VOL^6^**	67	H_2_O_2_	48	43	9
7	**VOL^7^**	69	H_2_O_2_	52	44	4
8	**VOL^8^**	65	H_2_O_2_	48	27	25
9	**VOL^9^**	59	H_2_O_2_	42	41	17
10	**VOL^10^**	66	H_2_O_2_	58	42	-
11	**VOL^1^**	85	TBHP	26	32	42
12	**VOL^2^**	88	TBHP	29	39	32
13	**VOL^3^**	80	TBHP	37	26	37
14	**VOL^4^**	87	TBHP	20	42	28
15	**VOL^5^**	82	TBHP	17	46	37
16	**VOL^6^**	84	TBHP	19	45	36
17	**VOL^7^**	83	TBHP	11	56	33
18	**VOL^8^**	84	TBHP	13	57	30
19	**VOL^9^**	85	TBHP	22	40	38
20	**VOL^10^**	82	TBHP	24	48	28

**Table 3:** Epoxidation of *S*(−)-limonene in the presence of oxidovanadium(V) Schiff base complexes as catalysts.

Entry	Catalyst	Yield (%)	Oxidant	Epoxide (%)	Diopoxide (%)
1	**VOL^1^**	23	H_2_O_2_	84	16
2	**VOL^3^**	25	H_2_O_2_	76	24
3	**VOL^9^**	19	H_2_O_2_	82	18
4	**VOL^1^**	59	TBHP	69	31
5	**VOL^2^**	71	TBHP	50	50
6	**VOL^3^**	67	TBHP	64	36
7	**VOL^4^**	66	TBHP	65	35
8	**VOL^5^**	62	TBHP	65	35
9	**VOL^6^**	76	TBHP	57	43
10	**VOL^7^**	72	TBHP	62	38
11	**VOL^8^**	63	TBHP	69	31
12	**VOL^9^**	55	TBHP	72	28
13	**VOL^10^**	58	TBHP	63	37

**Table 4:** Epoxidation of (−)-α-pinene in the presence of oxidovanadium(V) Schiff base complexes.

Entry	Catalyst	Yield (%)	Oxidant	(−)-α-Pinene Oxide (%)
1	**VOL^1^**	14	H_2_O_2_	86
2	**VOL^3^**	18	H_2_O_2_	88
3	**VOL^9^**	15	H_2_O_2_	81
4	**VOL^1^**	51	TBHP	61
5	**VOL^2^**	59	TBHP	73
6	**VOL^3^**	64	TBHP	59
7	**VOL^4^**	55	TBHP	63
8	**VOL^5^**	54	TBHP	67
9	**VOL^6^**	45	TBHP	66
10	**VOL^7^**	48	TBHP	49
11	**VOL^8^**	66	TBHP	62
12	**VOL^9^**	58	TBHP	59
13	**VOL^10^**	62	TBHP	70

## Data Availability

Not applicable.

## Supplementary Materials

The following are available online at https://www.mdpi.com/article/10.3390/molecules28217408/s1, Figure S1: The IR spectra of oxidovanadium(V) complexes.
