# Supplementary material for: Synthesis, Spectroscopic Characterization, Catalytic and Biological Activity of Oxidovanadium(V) Complexes with Chiral Tetradentate Schiff Bases
Source: Molecules. 2023 Nov 3;28(21):7408. doi: 10.3390/molecules28217408 (PMC10649191; doi:10.3390/molecules28217408)
Supplement: Supplementary file 1 [file molecules-28-07408-s001.zip › molecules-2663636-supplementary.pdf]

Figure S1. The IR spectra of oxidovanadium(V) complexes

VOL[1]

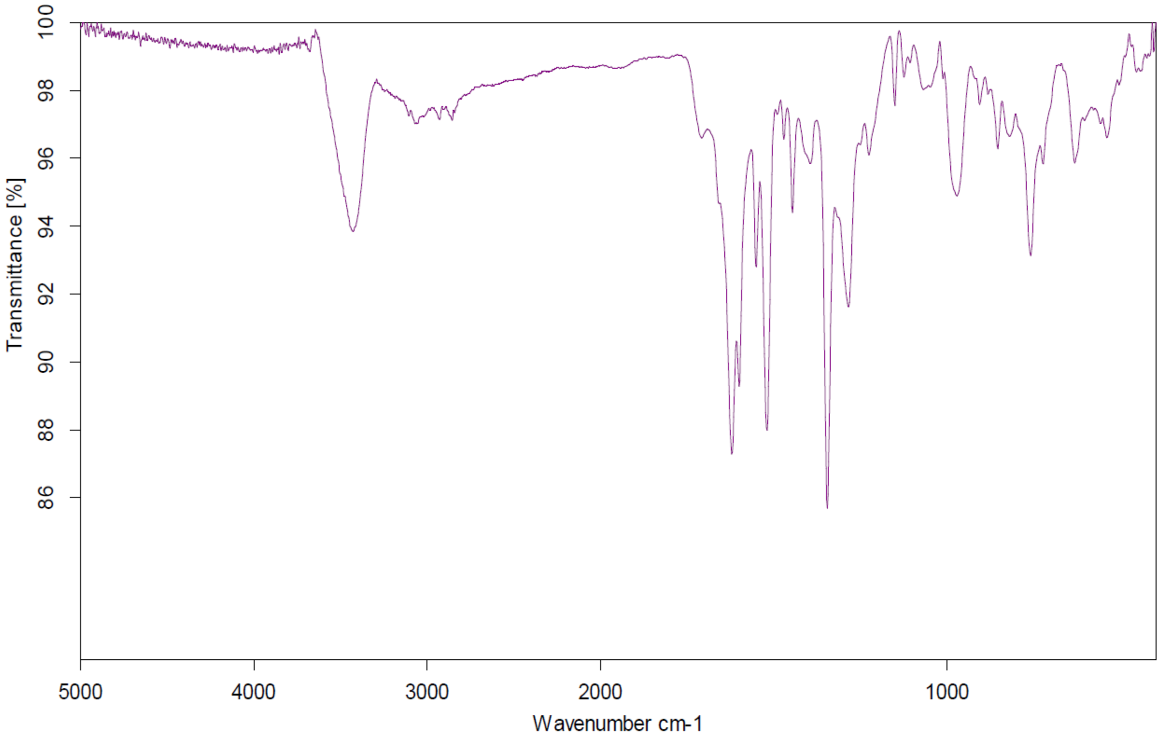

VOL[2]

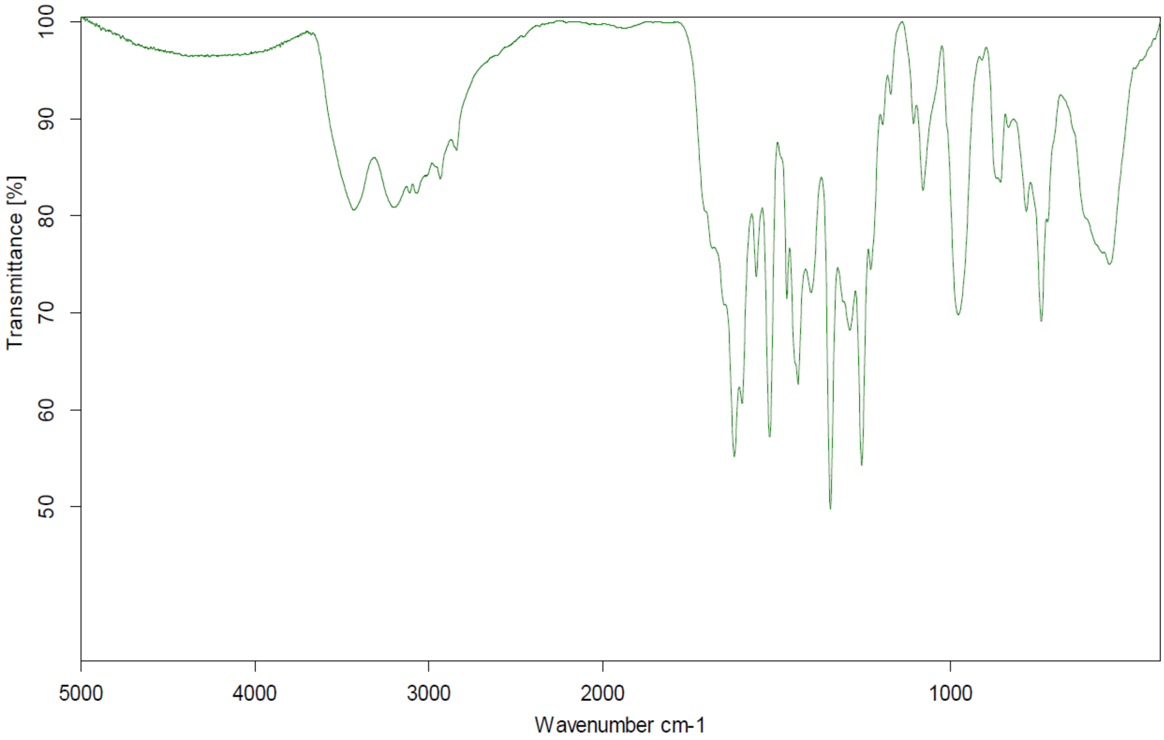

VOL[3]

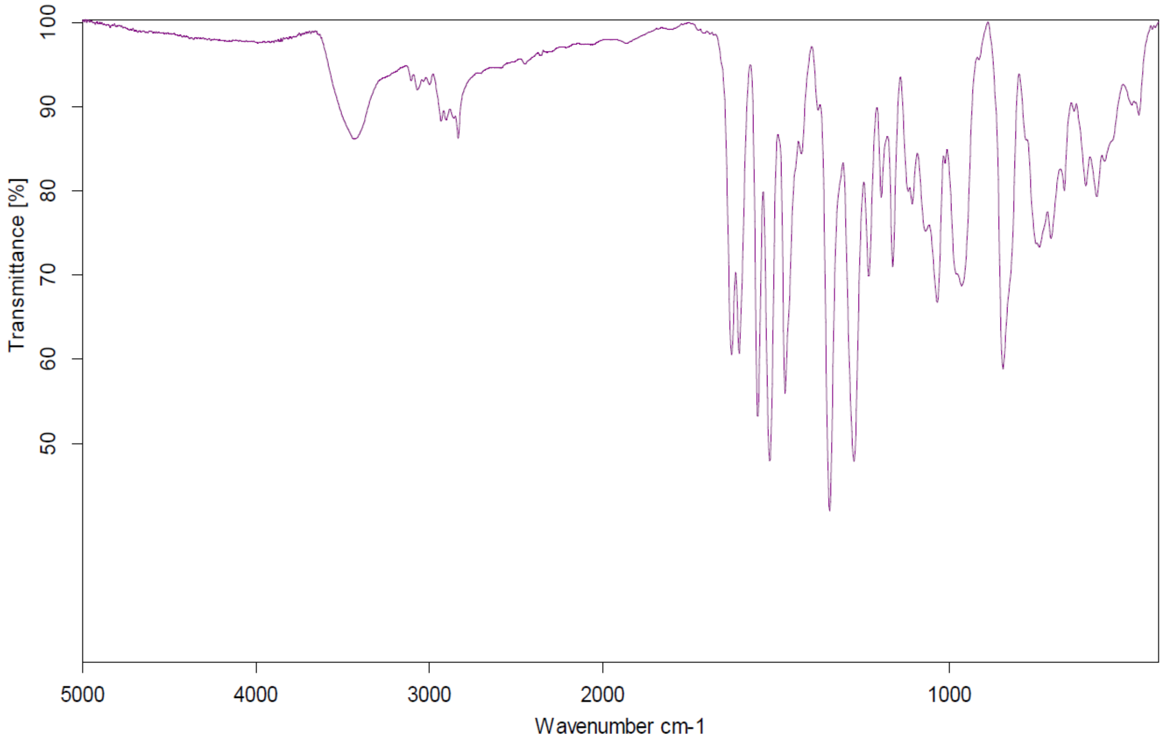

100
90
80
70
60
50
Transmittance [%]
5000
4000
3000
2000
1000
Wavenumber cm-1

VOL[4]

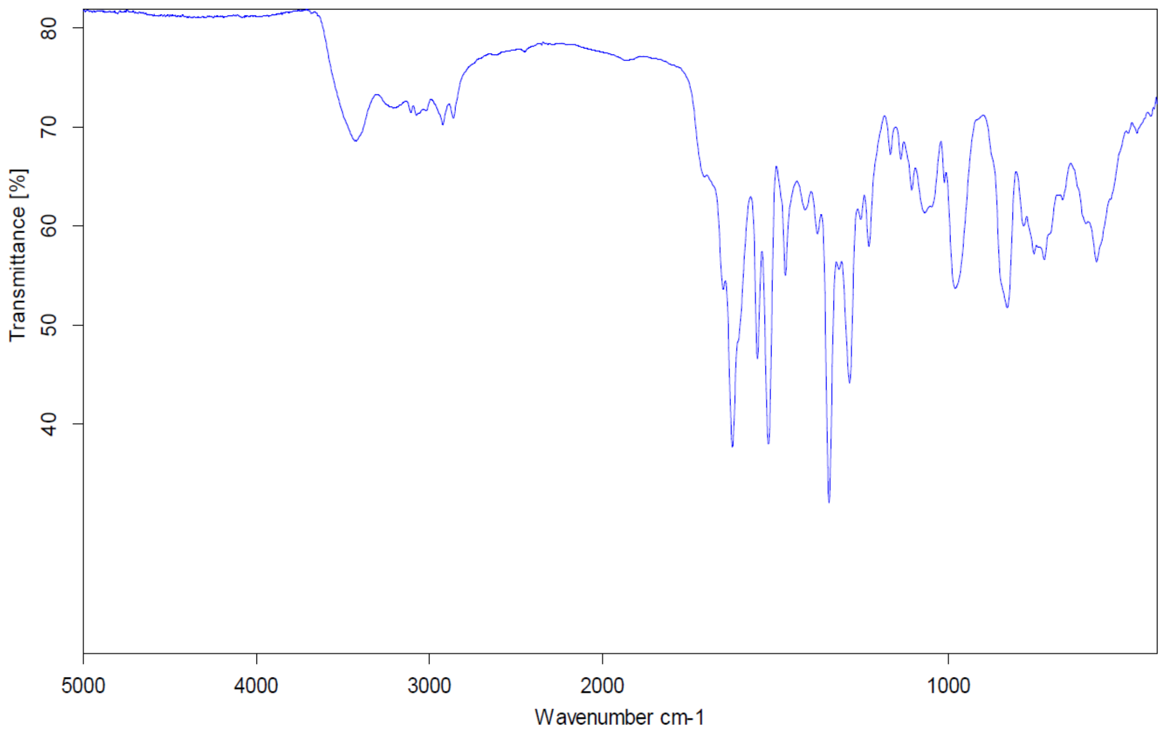

80
70
60
50
40
Transmittance [%]
5000
4000
3000
2000
1000
Wavenumber cm-1

VOL[5]

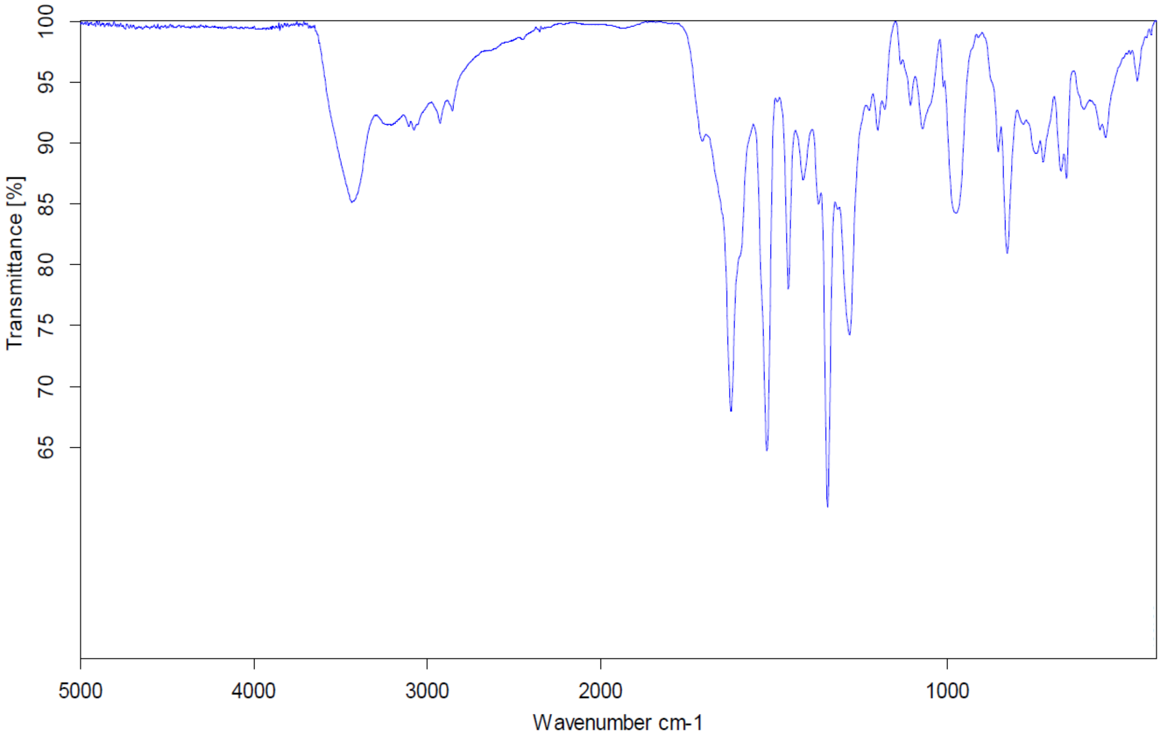

Transmittance [%]
100
95
90
85
80
75
70
65
5000
4000
3000
2000
1000
Wavenumber cm-1

VOL[6]

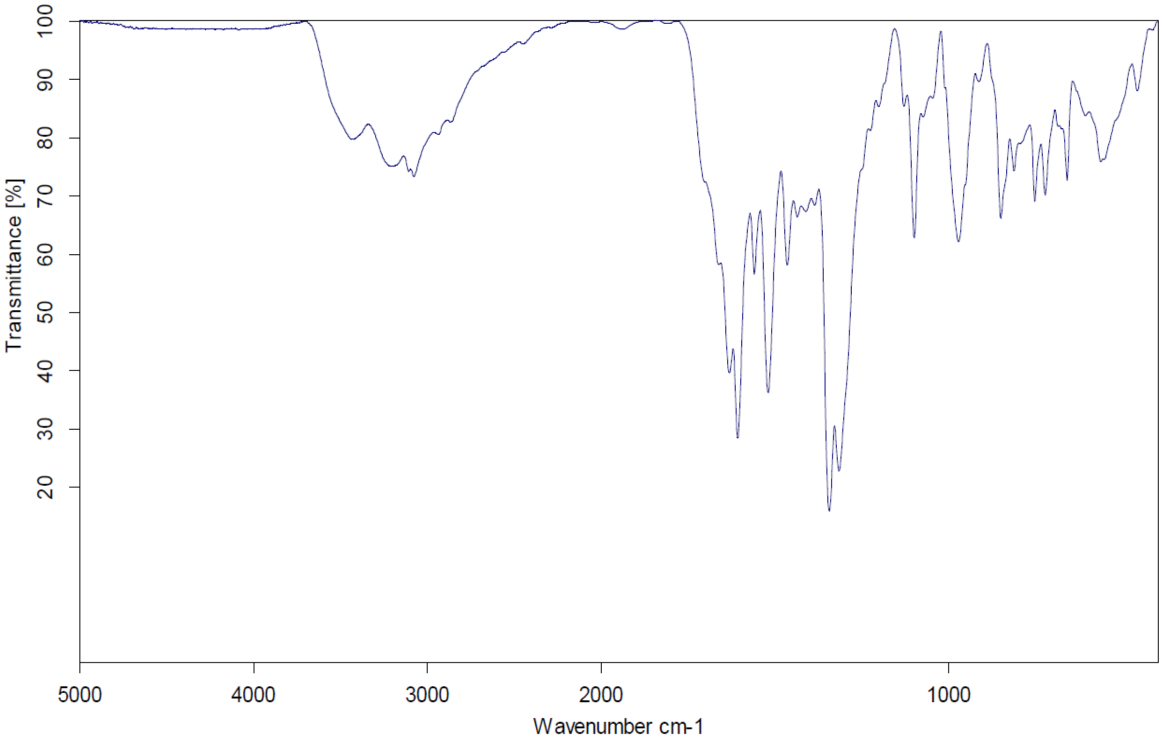

Transmittance [%]
100
90
80
70
60
50
40
30
20
5000
4000
3000
2000
1000
Wavenumber cm-1

VOL[7]

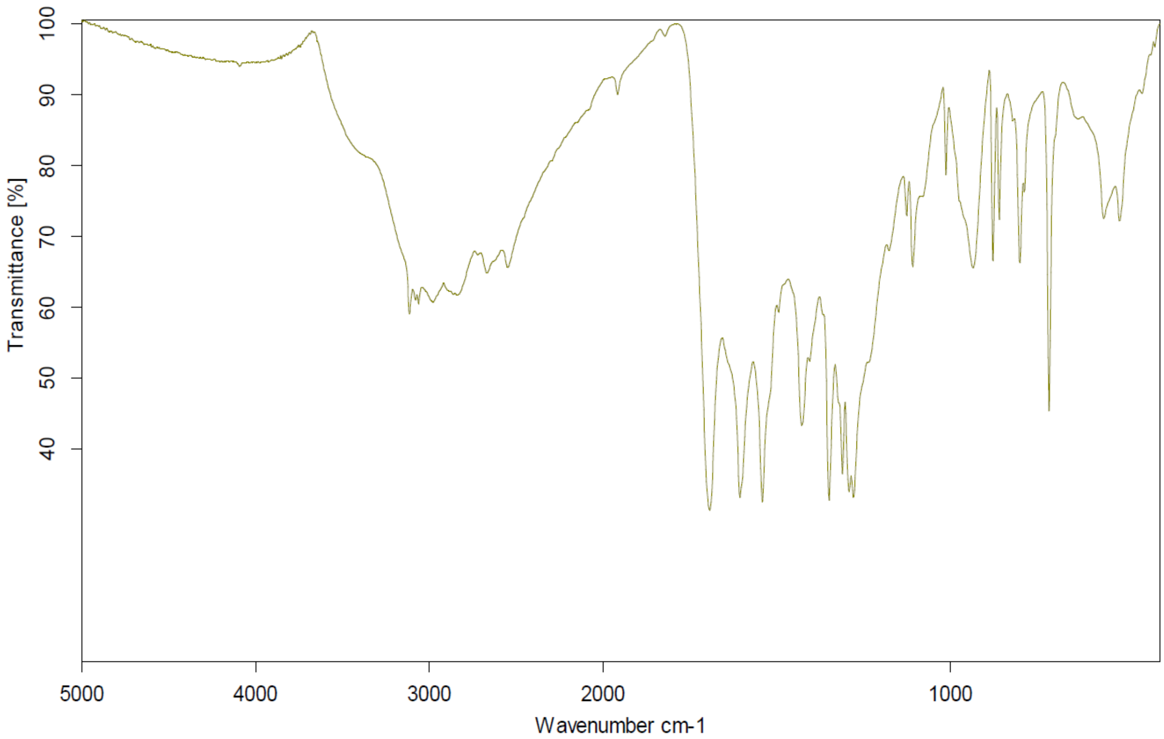

Transmittance [%]
100
90
80
70
60
50
40
5000
4000
3000
2000
1000
Wavenumber cm-1

VOL[8]

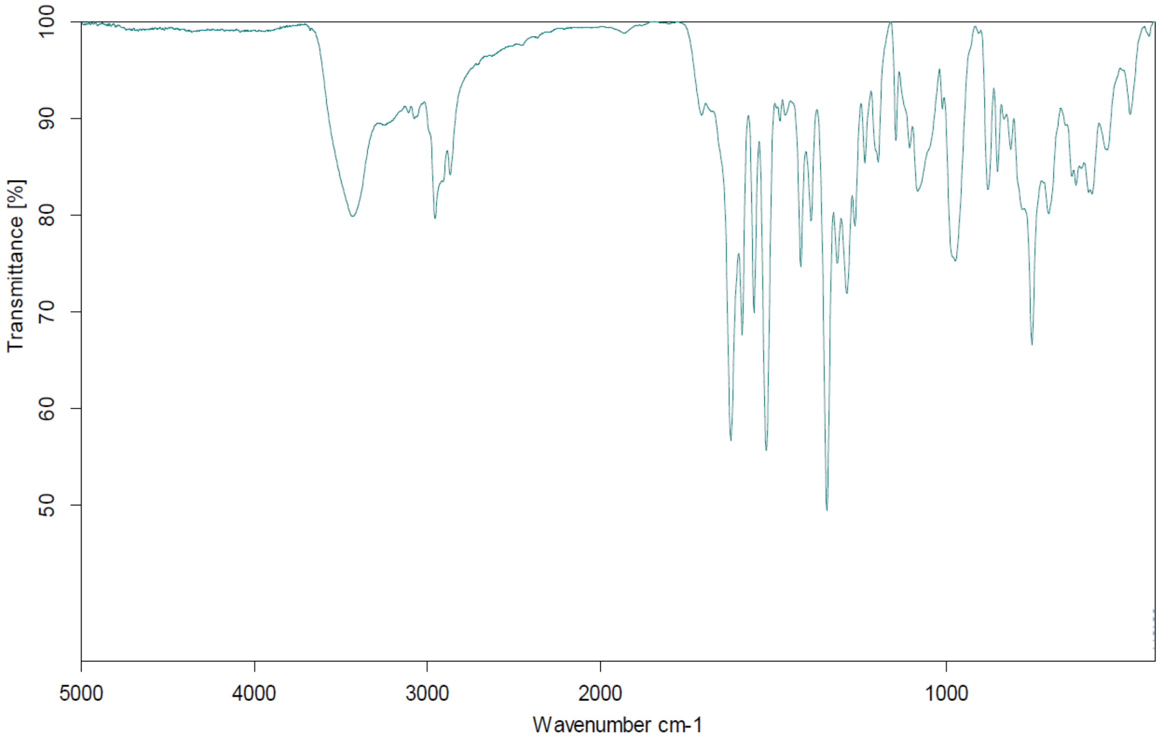

Transmittance [%]
100
90
80
70
60
50
5000
4000
3000
2000
1000
Wavenumber cm-1

VOL[9]

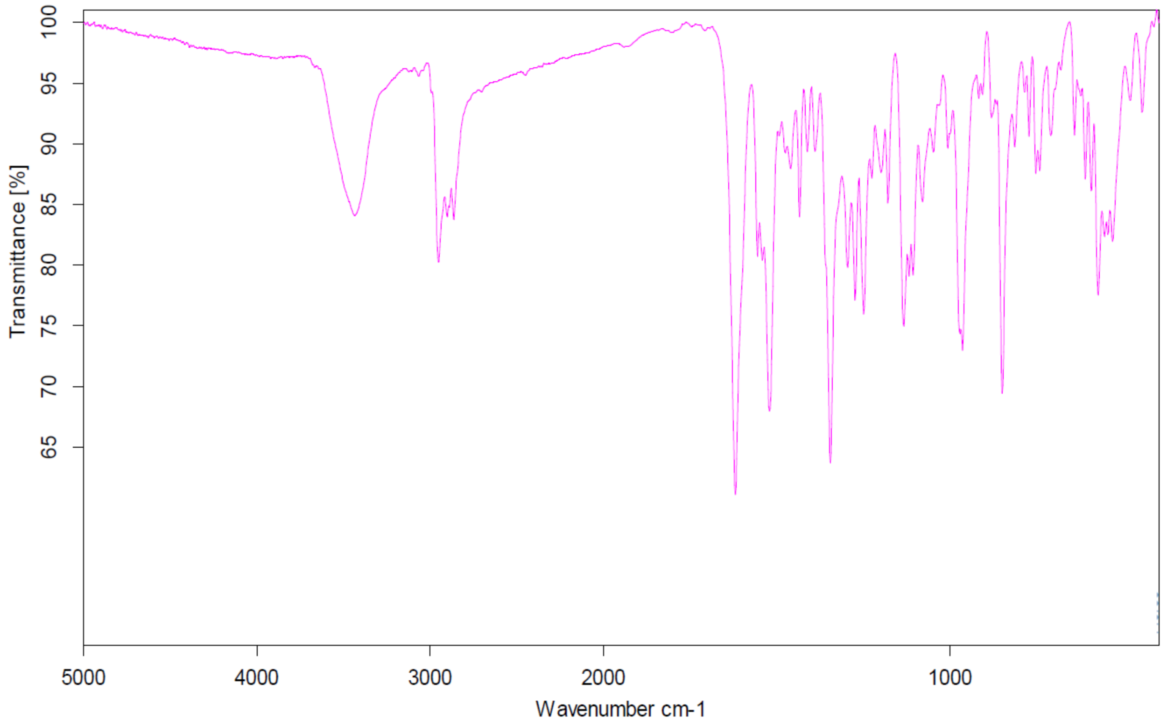
100
95
90
85
80
75
70
65
Transmittance [%]
5000
4000
3000
2000
1000
Wavenumber cm-1

VOL[10]

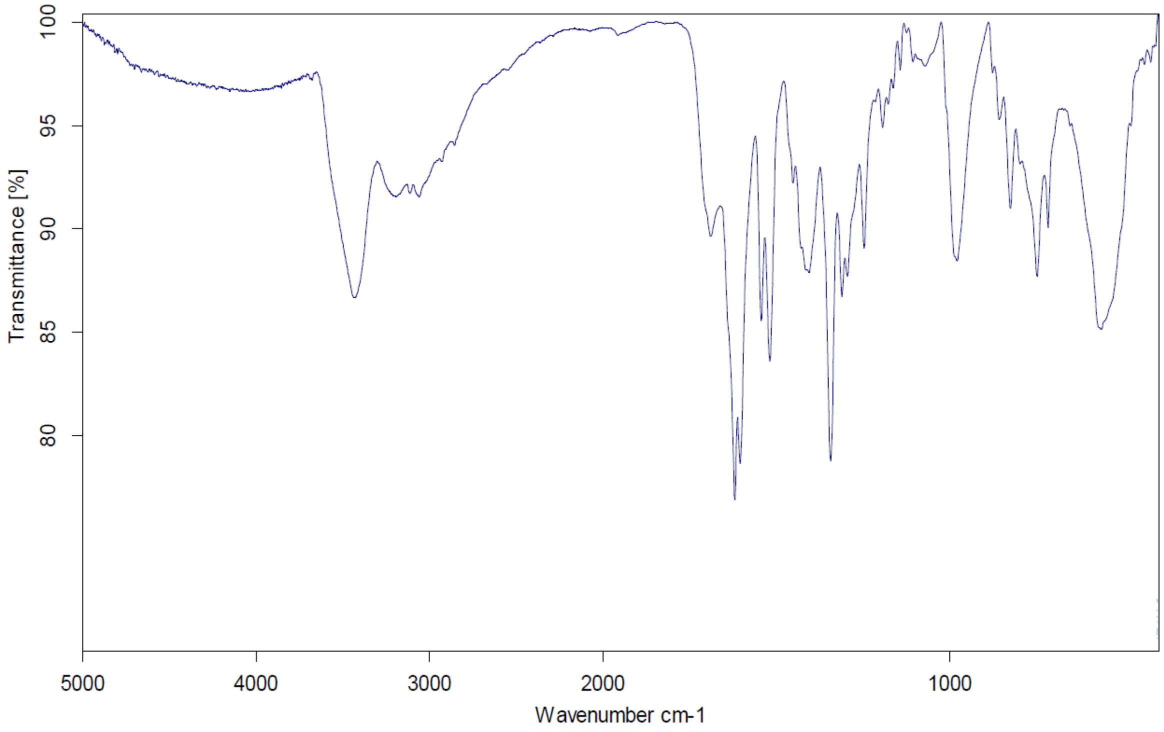
100
95
90
85
80
Transmittance [%]
5000
4000
3000
2000
1000
Wavenumber cm-1
